# Impact of intraspecific variation in insect microbiomes on host phenotype and evolution

**DOI:** 10.1038/s41396-023-01500-2

**Published:** 2023-09-02

**Authors:** Claudia Lange, Stéphane Boyer, T. Martijn Bezemer, Marie-Caroline Lefort, Manpreet K. Dhami, Eva Biggs, Ronny Groenteman, Simon V. Fowler, Quentin Paynter, Arletys M. Verdecia Mogena, Martin Kaltenpoth

**Affiliations:** 1 Manaaki Whenua Landcare Research, Lincoln, New Zealand; 2 Institut de Recherche sur la Biologie de l’Insecte, UMR 7261 CNRS - Université de Tours, Tours, France; 3 Above-Belowground Interactions Group, Institute of Biology, Leiden University, Leiden, The Netherlands; 4 UMR 7324 CITERES, Université de Tours, Tours, France; 5 Manaaki Whenua Landcare Research, Auckland, New Zealand; 6 Institute of Plant Sciences, University of Bern, Bern, Switzerland; 7 Department of Insect Symbiosis, Max Planck Institute for Chemical Ecology, Jena, Germany

**Keywords:** Microbial ecology, Symbiosis

## Abstract

Microbes can be an important source of phenotypic plasticity in insects. Insect physiology, behaviour, and ecology are influenced by individual variation in the microbial communities held within the insect gut, reproductive organs, bacteriome, and other tissues. It is becoming increasingly clear how important the insect microbiome is for insect fitness, expansion into novel ecological niches, and novel environments. These investigations have garnered heightened interest recently, yet a comprehensive understanding of how intraspecific variation in the assembly and function of these insect-associated microbial communities can shape the plasticity of insects is still lacking. Most research focuses on the core microbiome associated with a species of interest and ignores intraspecific variation. We argue that microbiome variation among insects can be an important driver of evolution, and we provide examples showing how such variation can influence fitness and health of insects, insect invasions, their persistence in new environments, and their responses to global environmental changes.

**Figure:**
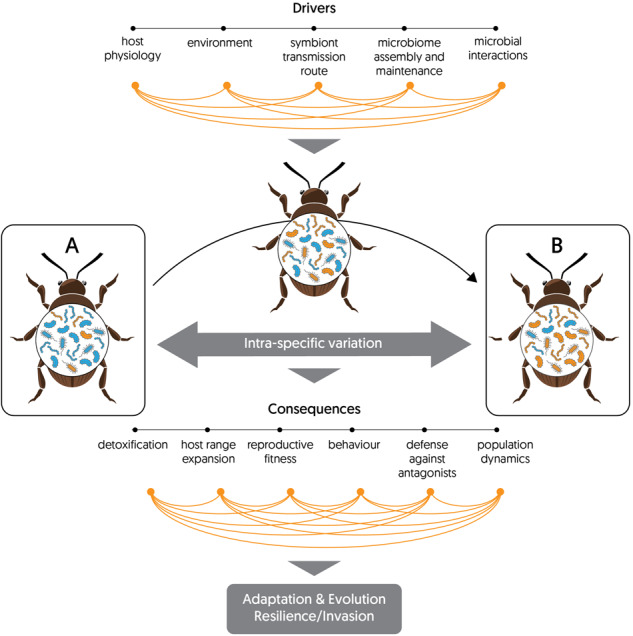
A and B are two stages of an individual or a population of the same species. The drivers lead to a shift in the insect associated microbial community, which has consequences for the host. The complex interplay of those consequences affects insect adaptation and evolution and influences insect population resilience or invasion.

A and B are two stages of an individual or a population of the same species. The drivers lead to a shift in the insect associated microbial community, which has consequences for the host. The complex interplay of those consequences affects insect adaptation and evolution and influences insect population resilience or invasion.

## Introduction

Insects are associated with a range of microbes that influence their biology and life history traits. The insect-associated microbial community (the microbiome) can vary between, but also within species [1]. Meta-analyses of factors contributing to insect microbiome structure and diversity across insect orders are rare. Host species and diet/trophy appear to be the most relevant drivers, but sex, life stage, and sample origin/habitat also have some impact, while the abundance of endosymbionts and phylogeny only have weak influence [2–6]. Intraspecific microbiome variation can be driven by several factors, such as the host itself, the diet, and the environment [7–9]. Microbe transmission routes, recruitment, maintenance, and interactions further shape these variations [10–12]. Insect symbionts can be separated into obligate (primary) and facultative (secondary) symbionts. While obligate symbionts are essential for their hosts’ survival and reproduction and usually have an ancient stable host association through vertical transmission, facultative symbionts are not required for growth or reproduction but can also affect adaptive host traits and can be horizontally transmitted [13]. In the context of intraspecific variation, facultative symbionts are of particular interest, and we focus this perspective on the importance of facultative insect symbionts that often vary in prevalence and abundance within and between insect populations. Intraspecific microbiome variation does not only have consequences for individual insects by impacting their behaviour, metabolism, and defence against antagonists, but also affects insect populations through changing reproduction, host range expansion, and host race formation [14]. Such population-level adaptations can have significant implications for insect invasions and population resilience and may ultimately drive evolution.

This perspective synthesises recent insights into how microbes control insect physiology and behaviour and describes the consequences of microbiome changes on insect invasions and persistence in novel ecosystems. We discuss the latest literature on the drivers and consequences of microbiome variation in insects with focus on herbivorous species, due to the available literature, and propose future research directions that are needed to improve our understanding of how intraspecific microbiome variation impacts host ecology and evolution. While there are many descriptive studies, experimental and field studies often ignore intraspecific microbiome variation, so its effect on host phenotypic traits, performance, and population dynamics remains poorly understood. This is particularly concerning given the potential of microbes to enable their insect hosts to rapidly adapt to changing environments, a topic that is highly relevant in the context of insect invasions and to understand the susceptibility or resilience of insect populations in the face of global environmental changes.

## Key drivers of intraspecific variation in microbial communities

### Insect host physiology

The host plays a key role in determining its microbial diversity, especially during development from immature to adult life stages, when insects undergo considerable morphological and physiological changes. This leads to diversification of ecological niches of larvae and adults, thereby reducing intraspecific competition [15]. Invertebrates can either preserve beneficial symbionts through life stages [16] or decouple microbial communities between larval and adult stage [17, 18]. As an example, dragonflies change their microbiome richness when they change from aquatic life in larval stages to terrestrial life in their adulthood [19]. A recent review summarised that the insect gut hosts the highest diversity of microbes across all invertebrates [7], providing a high potential for intraspecific variation. Guts shape microbial communities via chemical and physical conditions such as pH, nutrient availability, immune system, oxygen levels, and compartmentalisation. For example, the termite gut system has several compartments, and both pH and oxygen decrease from one compartment to another as food passes through. This enables the establishment of specialised bacteria in different compartments that help termites with cellulose degradation and nitrogen fixation [20]. While most studies on intraspecific microbiome variation focus on insect guts [1, 21, 22], microbes also inhabit other insect organs with specific physical and chemical properties, that can impact the assembly and functioning of microbiomes, such as haemolymph and salivary glands, especially in blood sucking insects [23]. In some species, the diversity in these organs can be higher than in the gut. For example, bacterial diversity is much higher in reproductive organs and in saliva of a number of different mosquito species than in the gut [24, 25], suggesting that there may be more intraspecific variation in microbiome composition in these organs than in the gut.

### External environmental factors

**Fig. 1:**
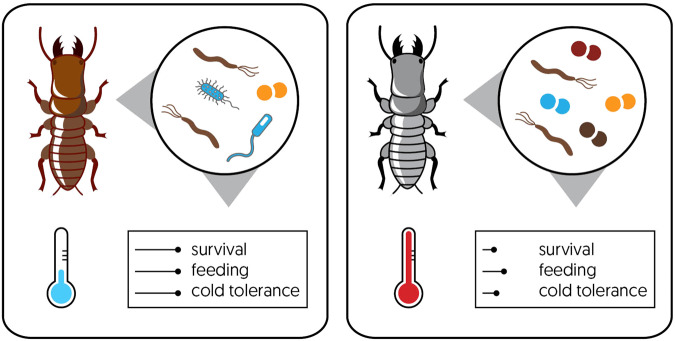
Warming reduces microbial community diversity and survival, feeding, and cold tolerance responses in termites. Experimental warming in Eastern subterranean termites (*Reticulitermes flavipes*) resulted in a reduction in gut prokaryotic diversity, especially when exposed to elevated temperature treatment (35 °C) (*p* < 0.05). The community composition also exhibited significant differences with Bacteroidetes symbionts increasing markedly under warming. Stress tolerance of termites also declined with a reduction in feeding, survival and cold tolerance responses observed [149]. While feeding activity and dispersal of termites is expected to rise under warming [150], gut dysbiosis due to warming may alter their survival and persistence.

In addition to the host physiology, external factors, or the environment in which the insects live, also have a major impact on their microbial communities. When insect microbiomes shift, the environment is a dominant source of the microbes that are acquired. Diet (food as an external environmental factor) is often mentioned as one of the main factors that influence the diversity of insect microbiomes, especially among herbivorous [8, 9] and carnivorous insect taxa [26]. The diet of an insect can act as a source of novel microbes when they are ingested with the food [27], but nutritional properties of the food can also influence an insect’s microbiome via its effect on the already existing microbes [28]. However, even though diet has a major influence on the composition or abundance of the microbes of an insect, several studies have shown that non-food aspects of the local habitat of the insect also act as important factors determining the microbiomes of insects. For example, the folivorous cabbage moth *Mamestra brassicae* actively acquires microbes from soil [29], and these microbes can be beneficial to its host as they may increase pesticide resistance [29–31]. The microbiome composition of insects can also be affected by environmental factors, such as temperature, habitat, elevation, and human interference [19, 32–34]. For example, increases in temperature lead to reduced microbial diversity and increased abundance of specific taxa associated with Eastern subterranean termites, which negatively impacts termite survival (Fig. 1).

### Symbiont transmission routes

Within the environment, insect-associated microbes are acquired and transmitted in several ways. The transmission route of intracellular symbionts is predominantly vertical as these symbionts can be present within reproductive cells or transferred inside the developing egg. However, cases of horizontal acquisition have also been documented, for example in the whitefly *Bemisia tabaci* subjected to non-lethal probing by *Wolbachia*-infected parasitoids [35]. To survive and develop, individuals also need specific gut microbial communities from the onset of their lives [36]. Despite being extracellular, these microbes can still be vertically transmitted, through the smearing of the egg surface [37], the inoculation of the oviposition sites with faeces, specific secretions [38], or more sophisticated structures that are produced by females [39, 40] and consumed by the offspring immediately after hatching from the egg. Young individuals of gregarious and social insects can obtain the necessary symbionts by feeding on the faeces of their congeners (coprophagy) [41] or by direct fluid exchange from anus to mouth (proctodeal trophallaxis) [42]. The transmission of gut microbiota can also follow a horizontal route, either mediated by soil or plant materials that have been externally smeared with the faeces of other individuals [43], or through trophic interactions with other species [11]. Transmission may occur, for example, when two herbivores feed on the same plant [44], when predators feed on their prey [45], or when parasitoids feed on their hosts [46]. The recruitment of extracellular gut bacteria invariably relies on their ingestion and their colonisation of the gut lumen [20]. It has been hypothesised that specific traits can also be acquired from transient bacteria (such as plant-associated bacteria) that do not establish in the gut but engage in horizontal transfer with native bacteria that are already established in the gut [47]. Both pathways, horizontal and imprecise vertical transmission of insect-associated microbes, can promote intraspecific microbiome variation and stochasticity [11, 44, 48].

### Microbial assembly and maintenance

Once the transmission of microbes has occurred, different processes affect how the community gets assembled and maintained. The processes of microbial community assembly and maintenance affect the function of the microbiome and host fitness. For example, a reciprocal microbiome transplant in the Water flea *Daphnia magna* found that different host genotypes selected different microbiomes. Such selective uptake of specific microbes with host beneficial functions as well as a high diversity of strains with complementary gene functions increased the host’s tolerance to toxic cyanobacteria [49]. In the whitefly *Bemisia tabaci*, the acquisition of gut-associated bacteria was strongly affected by the identity of the host plant. After switching the host plant, host-specific microbiomes were assembled and maintained over multiple generations, leading to improved metabolism and survival of the host. This was largely attributed to direct effects of available nutrients and or secondary metabolites [12]. An example for local adaptive microbiome maintenance is the Colorado potato beetle (*Leptinotarsa decemlineata*), whose microbiome adapted along its invasion path in China. The beetle population that was leading the invasion front had a higher abundance of microbiota in oral secretions, a higher gut bacterial diversity, and different relative abundance than the ancestral population 500 km away. The adapted microbiome improved the suppression of plant-induced defences and enabled geographic expansion [50].

### Microbial interactions

Individual members of the microbiome also interact with each other, which affects the microbiome composition and host performance. For example, under nutritional stress, the *Drosophila* symbionts *Lactobacillus plantarum* and *Acetobacter pomorum* exchange metabolites to fulfil their own requirements. The provisioning of lactate from *L. plantarum* to *A. pomorum* supports this species’ metabolism and results in a release of anabolic metabolites by *A. pomorum*, which in turn, supports host larval growth of individuals that are exposed to nutritional stress [51]. In contrast, negative interactions between symbionts, such as competition or antagonism also occur. The silk moth *Bombyx mori* is protected from microsporidia pathogens by its symbiont *Enterococcus faecalis* that reduces spore germination, ameliorates gut injury, and reduces colonisation of the pathogen. Increasing abundance of *E. faecalis* reduces the abundance and infection efficiency of the pathogen [31]. However, in the honeybee gut, closely related *Lactobacillus* species are able to overcome competition for nutritional resources and to coexist because they utilise different pollen-derived carbohydrates. This suggests that dietary choices of the host or natural variation of the diet will influence the gut microbiome diversity [52]. In the bean bug *Riptortus pedestris*, *Burkholderia* symbionts improve growth and fecundity of the host by recycling host metabolic wastes in the midgut crypts. Upon acquisition of the symbiont from soil, the acquisition of other symbionts is stopped by it altering the host midgut morphology, a mechanism that proposedly supports host-symbiont specificity in the absence of vertical transmission [53].

## Consequences of intraspecific variation in microbial communities for the host

Although evidence exists for intraspecific variation in insect microbiomes and for the various drivers, the functional implications of these changes for the host and for host evolution remain poorly understood. In the following, we discuss several examples of consequences for the insect host.

### Insect behaviour

Microbes can shape how insects respond to a stimulus [54]. Although most of the studies addressing this focus on the implications of specific symbionts, entire microbial populations are also linked to diverse roles in the physiology and behaviour of insects such as *Drosophila*. For instance, microbial composition is linked to the dietary preference of *Drosophila melanogaster* [55]. In this study, the insects were more attracted towards the diet that contained the microbes on which they were reared, implying that the gustatory preference can be driven by microbial populations. An example of a tripartite symbiosis that affects insect behaviour is the “killer yeast”. When *Drosophila* spp. associated *Saccharomyces cerevisiae* strains are infected with two complementary viruses they turn into killer yeast strains. These strains kill uninfected yeasts in fruit and are more attractive to insect vectors. This interaction influences the feeding of *Drosophila* spp. and promotes the dispersal of killer yeasts to new fruits [56].

Microbes are also widely associated with the capacity of insects to find suitable egg-laying spots. This phenomenon has been described for flies and other insects such as *Encarsia pergandiella* [57]. This parasitoid wasp changes its oviposition habits when invaded by *Cardinium* spp., enabling the bacterium to manipulate host behaviour and to spread through the insect population [58].

### Metabolism and detoxification

For plant feeding insects, the microbial community is often critical for their nutritional status and survival [59]. The relevance of the obligate symbionts for nutrient provisioning (such as essential amino acids, vitamins, and sterols), digestion (such as plant cell wall degrading enzymes), and detoxification has remained a focal research theme. For example, some symbionts are involved in the physiological mechanisms of sterol intake by different insect species [60]. Nevertheless, studies of obligate symbionts do not show intraspecific variation, and the contribution of facultative symbionts to metabolism, digestion, and detoxification just recently started to garner interest [47, 61, 62]. The insect digestive system shelters a plethora of microbes, whose role in degrading plant structural compounds, providing nutrients, and detoxifying plant secondary metabolites has been, although less, already acknowledged [27, 47]. Gut symbionts are in contact with environmental microbes, increasing the possibilities of genetic material transmission or even the substitution by new microbes. Hence, the insect host could attain new detoxifying genes or perhaps microbes with novel metabolic capacities. Some studies, including a field experiment, concluded that the capacity of some insects to feed on a wide range of plants is moderately related to the facultative microbial associates [12]. Moreover, insect gut microbes have been reported to metabolise terpenes, flavonoids, alkaloids, phenolics, and isothiocyanates [63–68]. Microbes associated with saliva of herbivorous insects can influence defence responses in the host plant [69, 70], which influences the quality of the ingested food and, in turn, the gut microbiome. However, how microbes from saliva vary in their effects on induced plant defence, plant interactions, and the role of intraspecific variation in saliva microbiomes is poorly understood.

### Defence against antagonists

By stimulating the host’s immune system or by directly inhibiting or competitively excluding antagonists, microbial symbionts can contribute to the defence of their host against antagonists, including predators, parasitoids, parasites, and pathogens [71]. In contrast to nutritional symbioses, defensive insect-microbe partnerships are often dynamic and experience horizontal influx of symbionts, resulting in intraspecific differences in microbial communities that can significantly impact defence traits of the insect host [71].

In aphids, facultative endosymbionts can enhance protection of their hosts against parasitoid wasps [72–78], fungal pathogens [79, 80], and viruses [81]. However, these defensive symbionts incur costs for the insect in the absence of the relevant antagonists [73, 82–85]. Furthermore, the extent of protection (at least against parasitoids) is governed by the interaction between host, symbiont, and antagonist genotype, resulting in complex fitness outcomes of symbiont infection for the host that are dependent on antagonist abundance in the environment [85, 86]. As such, symbiont-mediated protection can have cascading effects on multitrophic communities and affect species coexistence under laboratory conditions [74, 87]. Field studies support a defensive role of hemipteran facultative symbionts against antagonists but yield mixed results on fitness consequences for the host [82, 88, 89]. Thus, further studies are needed to understand how intraspecific differences in host-symbiont-antagonist interactions affect population dynamics and species coexistence in the field [90].

Other insects also engage in defensive symbiotic associations with bacterial or fungal partners, but evidence for intraspecific variation in symbiont-mediated protection and its fitness consequences remains patchy. Antibiotic producing bacterial symbionts of *Lagria* beetles [16, 91, 92] and beewolf wasps [93–95] predominantly rely on vertical transmission but experience occasional horizontal symbiont acquisition, resulting in multipartite defensive communities or occasional symbiont replacement, respectively [94, 96, 97]. While this has direct consequences for the defensive chemistry provided to the host [91, 92], the extent of this variation under field conditions and its relevance for host fitness remain to be explored. In a rare case of combining laboratory- and field-based investigations of a defensive symbiosis, it was found that *Spiroplasma* recently spread across *Drosophila neotestacea* populations in North America due to its role in protecting the host against the sterilising effects of a parasitic nematode [98]. Other studies revealing a protective role of gut bacteria against intestinal parasites [99] and cuticular microbes against pathogenic fungi [26, 100] indicate that symbiont mediated defence is common across insects. As intestinal and cuticular microbiomes are often variable in their composition, these studies also suggest an impact of intraspecific microbiome variation on host defence. However, systematic studies are urgently needed to characterise the extent of intraspecific variation in defensive microbial communities and their importance for host fitness and population dynamics under natural conditions.

### Host range expansion and host race formation

Gut microbiota can be an important driving force of the speciation process in phytophagous insects. Microbes play significant roles in the exploitation of a novel host plant by phytophagous insects [63, 68]. Indeed, the presence of a specific set of core microbes, capable of metabolising plant defence chemical compounds could explain the ability to exploit new hosts [47], which constitutes an essential first step towards host range expansion [101, 102]. However, the mechanisms underlying this phenotypic plasticity are still to be clarified [103].

It was recently hypothesised that rapid host plant switching might partially rely on transient associations between insects and bacteria, the latter providing an additional flexible metabolic “toolbox” that facilitates the effective use of a novel host plant [12]. By influencing the ability of the insect to feed on particular plant hosts, intraspecific variation of gut microbiota profiles can create ecological barriers that facilitate sympatric speciation. In addition to this symbiont-mediated niche exploitation and behavioural change (pre-mating isolation), symbiont-mediated intraspecific incompatibilities and coevolutionary processes (post-mating isolation) can also contribute to sympatric speciation [104]. Hence, intraspecific variations in insect-microbe associations could lead successively to host range expansion, host shift, host race formation, and ultimately to sympatric speciation [14, 105]. The entire sympatric speciation continuum must be considered to fully understand the role of insect microbiota variations in the speciation process of phytophagous insects. For example, even though changes in insect gut microbiome can happen over a short time frame [106], multi-generational studies [107] are essential to determine whether even transient insect-microbial associations enable the insect to explore a new host plant range, thereby providing future ecological and evolutionary potential.

Many hypotheses have been proposed regarding the impact of transient and horizontally acquired microbes during host range expansion and host shift events [12], however, experimental testing of these hypotheses is scarce. Similarly, while an extensive descriptive literature is available on the microbial composition of different populations or host races [108, 109], evolutionary studies focusing on the mechanisms underlying the acquisition and maintenance of microbial communities are still limited [110–112].

### Sexual communication, mate choice, and reproduction

Success of an insect population or species relies crucially on its ability to reproduce. Reproductive success depends on a range of phenotypic traits, including fecundity (gamete production and survival), rate of reproduction, and successful mating. Mating success is affected by mediation of intraspecific communication [113], specificity of mating behaviours, and mate selection.

Evidence of microbial influence on reproductive fitness and associated traits and behaviours has been accumulating across a range of insect hosts. In bed bugs (*Cimex lectularis*), sexually transmitted commensal bacteria can increase sperm mortality and negatively influence fecundity [114]. Egg production and larval development in mosquitoes (*Aedes aegypti*) is reduced in the absence of its symbiotic bacteria community, an effect that was rescued following reintroduction of key bacterial species—*Serratia* sp. and *Elizabethkingia* sp. [115, 116]. A gene of the symbiotic double-stranded RNA virus of *Drosophila biauraria* encodes a male-killing protein. The gene may have been acquired by the virus through shuffling of genomic segments, or reassortment, and may provide opportunities for intraspecific variation in insect microbiomes affecting reproduction [117]. On the other extreme, females of the parasitic wasp *Asobara tabida* become incapable of oogenesis when *Wolbachia-*free [118], with intraspecific variation in *Wolbachia* strains committing variable impacts on oogenesis and cytoplasmic incompatibility [119]. These examples highlight the differential impacts of microbial partners on insect host fecundity.

**Fig. 2:**
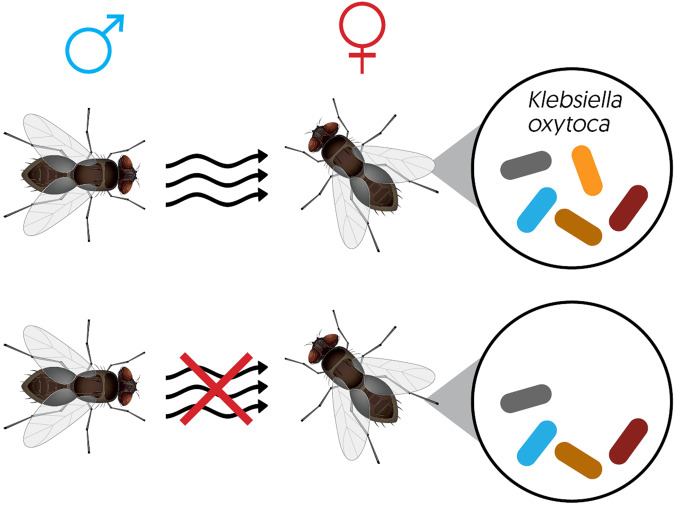
Vertically transmitted *Klebsiella oxytoca* influences Oriental fruit fly mate selection. The Oriental fruit fly (*Bactrocera dorsalis*) harbours a diversity of gut symbionts, of which vertically transmitted Enterobacteriaceae bacteria *Klebsiella oxytoca* are reported to enhance mate selection [151]. In the absence of this symbiont, significant reduction in mate attraction (via olfaction, *p* < 0.0001) and mating outcome (via sperm deposition, *p* < 0.0001) for gnotobiotic virgin females has been reported [152]. Subsequently reinfected with this symbiont, virgin female flies regain both mate attraction and sperm accumulation responses from male flies [152]. By increasing the likelihood of successful mating, *K. oxytoca* along with other symbionts can facilitate the invasion success of the Oriental fruit fly.

Mate signalling and choice assays in fruit flies have revealed intricate interactions with microbes associated with gut and reproductive organs. *Bacillus* spp. and other related bacteria localised in the rectum of *B. dorsalis* males can produce its sex pheromones, tri- and tetra-methylpyrazine [120]. Bacterial origin of the female sex hormone has also been reported in grass grub beetle (*Costelytra zealandica*), as a breakdown product of tyrosine by colleterial gland resident bacteria *Morganella morgani* [121, 122]. Attraction and mate selection in a variety of tephritid fruit fly species is also enhanced in the presence of specific bacterial symbionts [123] (Fig. 2).

Finally, microbial mediation of insect communication via pheromones and semiochemicals has received renewed interest recently [113, 124]. Broadly, bacteria can produce a remarkable variety of compounds that interfere with insect-insect communications as well as affect insect behavioural outcomes [124].

## Consequences for adaptation and evolution

Any changes to insect host biology have the potential to affect the population structure and dynamics. The right combination of microbial partners may support a resilient population, while a shift can contribute to population decline or invasion. Microbiome variation may be an adaptive trait, subject to natural selection, that increases host fitness according to a model on imprecise vertical transmission [48]. By extending phenotypic plasticity of the host, microbes can enable populations to be more flexible in changing environments, which ultimately affects adaptation and evolution.

### Implications for biodiversity, conservation, and biosecurity

Microbiomes change when arthropods encounter new or restrictive environments. Population size bottlenecks, common in conservation efforts, and biocontrol programmes, can result in loss of microbiome diversity [125], and consequently in reduced host fitness [126]. Captive rearing alone can result in loss or gain of harmless and beneficial microbes that then cause reductions in host fitness on the return of host populations to the wild [96, 127]. By conferring resistance to parasitoids in aphids, defensive symbionts can pose a serious challenge to the development of biological control agents [128, 129].

In the wild, could transfer of microbes between introduced host species and related indigenous host species cause unwanted changes, for example in host ranges of herbivorous arthropods? Rare cases of unpredicted non-target attack by introduced weed biocontrol agents all had explanations that were not microbiome-associated [130]. However, host shifts have been associated with microbiome change in a moth pest [107] and were created experimentally across generations in an aphid [131]. The potential contribution of microbes to insect invasions is exemplified by the hypothesis that a swap between pest and non-pest symbiont genotypes or a symbiont mix during host hybridisation led to the plataspid stink bug *Megacopta cribraria* becoming invasive in the USA [132]. Can microbiome manipulations also have conservation benefits? For example, the threatened Australian butterfly, *Ornithoptera richmondia* oviposits on an introduced invasive weed *Aristolochia littoralis* on which its larvae die [133]. Can we transfer microbiome components from butterfly species that naturally feed on *A. littoralis* in the weeds’ native range to the threatened Australian butterfly, turning an “ecological trap” plant into a host plant (and potentially assisting in native biocontrol of the weed)? Ultimately, understanding and manipulating arthropod microbiomes may allow us to reduce biosecurity risks, improve performance of beneficial arthropods, and enhance conservation of rare indigenous arthropods.

## Outstanding questions and future research directions

Current challenges in microbiome research are to go beyond descriptive studies towards functional analysis at lower taxonomic levels (within phyla, families, genera, and species) of microbe-microbe and microbe-host interactions, community effects and the targeted manipulation of microbiomes [1, 7, 8]. This requires measuring and modelling the resilience of host-associated microbial communities in non-model species in the field to generate quantifiable data [11, 134, 135]. Apart from better understanding the drivers and consequences of insect microbiome changes, a major challenge for future research is to better understand how to influence and manage microbiomes of insects [30, 136–141]. We can take advantage of methods that have been developed for human, plant or soil microbiomes and adopt them to improve the analyses of insect microbiomes [30, 142–144]. We selected several examples of novel methods that can further elucidate the complex interactions within insect microbiomes and how to manipulate them (Table 1). For example, altering the microbiome composition of insect pests may reduce the severity of the damage they inflict, or honeybees may become more resilient to climate change through altering the gut biome. As there are many drivers of insect microbiome composition, tractable manipulation in the field remains a major challenge [140]. We propose that the most immediate opportunities for microbiome management are therefore with insect species that are reared under controlled conditions and then released, like for biological control of pests such as predators or parasitoids, or herbivores used to control weeds. Improving their efficiency, for example via the introduction of symbionts that enhance fecundity or longevity during rearing, can have far reaching consequences, and this is in urgent need of investigation.

**Table 1:** Novel methods to study intraspecific variation in microbial communities of insects.

Method	Description	Applications	Benefits	Challenges	Reference
Paratransgenesis of obligate symbionts	Symbiont-mediated RNAi to silence host genes or genes of other symbionts	Studying symbiosis Preventing insects from vectoring pathogens Controlling pest insects	Improved field safety because obligate symbionts are less likely to colonise other insects than facultative symbionts	Technology to cultivate many obligates in axenic culture Engineering obligates in situ Social and regulatory acceptance	[136, 138, 139, 141]
Host-mediated symbiont selection	Guiding the host to acquire and maintain microbial components via exposure to specific conditions, such as stress	Microbiome manipulation Improving host resilience Controlling pest insects Replacing the method of artificially adding or removing taxa	Microbial communities that are adapted to the selected conditions and create the desired host effect Microbiome stability and resilience	Potential for off-target or undesirable side effects on host fitness and microbial community dynamics Translation from plant to insect microbiomes	[30, 137, 140]
Co-occurrence network analysis	Assessing relationships across diverse and complex microbial communities	Studying organisation and structure of communities Identification of keystone species Identification of factors that determine community structure Inferring taxa interactions	Observing the structure and function of diverse communities in situ	Interpretation of results Over- or underestimation of the true complexity Need for complementary experimental studies to confirm functions and interactions Translation from soil to insect microbiomes	[135, 145–147]
Microbiome association studies	Linking microbiome analyses to phenotypic descriptions	Studying symbiosis Identification of mechanisms that connect microbial community features to specific host traits	Observing the structure and function of diverse communities in situ	Translation from human or soil to insect microbiomes and ecological settings Biological confirmation of computational results Careful experimental design and robust analysis and data interpretation	[142–144]

We highlight the following four questions to be addressed to improve the understanding of intraspecific changes in insect microbiomes and the management of insect resilience or invasion:

1. What determines the functional resilience of host-associated microbial communities? While the core microbiome represents the most common and most abundant taxa, species with rare occurrence and low abundance can also be important for the functional stability and adaptation of the microbiome. These community members may be transient, but can supply functional redundancy, which may be critical under stressful conditions [12]. Learning more about the functions of individual species or strains, and the interaction networks within the microbiome will improve our understanding of resilience of microbiome functions [135, 145–147].
2. How can we achieve targeted manipulation of insect microbiomes for specific purposes (“microbiome engineering”), such as pest and pathogen control and biocontrol? The experimental design for host-mediated symbiont selection needs to be optimised. We need to develop tools to cultivate obligate symbionts in axenic culture to then engineer them or to engineer obligate symbionts in situ [136, 138, 139, 141, 148]. We also need to understand more about the complex functional relationships between symbionts and hosts and other host-associated organisms, such as plants and parasitoids to manipulate microbiomes successfully and safely.
3. How can we quantify microbiota switches that naturally occur in insects? To study the microbiome shift within populations, we need to assess the prevalence and abundance of taxa through time in the field. This will give us the rate of microbiome change in real-world scenarios, rather than anecdotal descriptions.
4. How do microbiome-affected processes, such as host range and mate selection influence each other? These are complex interactions, leading to species-specific outcomes that need to be studied with careful consideration for selected conditions. To answer this question, we cannot rely on results for model organisms but need to directly investigate the species and systems in question.

## Conclusion

The multiplicity of transmission routes and sources of variation in insect microbiota have been well identified. However, the extent of microbial shifts occurring through these different routes remains poorly known. In addition to naturally occurring exchanges among sympatric species, the continuous increase in invasive species introductions may open the door to the transmission of new microbial symbionts, horizontally, between introduced and native species. As opposed to new abilities that might arise from “random” genetic mutations, the horizontal acquisition of new microbial species and the abilities they confer can be immediate. If adaptive, these abilities could spread through the population very rapidly, with significant consequences on the insect and its ecosystem. Consequences may include the ability for phytophagous insects to exploit new host plants (and cause greater plant damage), or the ability to cope with environmental stress (such as climate change), evade natural enemies or withstand diseases. A better fundamental understanding of intraspecific microbiome dynamics will improve the protection of insect biodiversity and the management of invasive insects, which will benefit the environment as well as local and regional economies.

## Data Availability

No datasets were generated or analysed during the current study.
